# Exchange biased surface acoustic wave magnetic field sensors

**DOI:** 10.1038/s41598-023-35525-6

**Published:** 2023-05-25

**Authors:** Viktor Schell, Elizaveta Spetzler, Niklas Wolff, Lars Bumke, Lorenz Kienle, Jeffrey McCord, Eckhard Quandt, Dirk Meyners

**Affiliations:** 1 Inorganic Functional Materials, Institute for Materials Science, Christian-Albrechts-Universität zu Kiel, 24143 Kiel, Germany; 2 Nanoscale Magnetic Materials – Magnetic Domains, Institute for Materials Science, Christian-Albrechts-Universität zu Kiel, 24143 Kiel, Germany; 3 Synthesis and Real Structure, Institute for Materials Science, Christian-Albrechts-Universität zu Kiel, 24143 Kiel, Germany

**Keywords:** Materials for devices, Sensors and biosensors, Magnetic properties and materials, Materials science, Magnetic properties and materials

## Abstract

Magnetoelastic composites which use surface acoustic waves show great potential as sensors of low frequency and very low amplitude magnetic fields. While these sensors already provide adequate frequency bandwidth for most applications, their detectability has found its limitation in the low frequency noise generated by the magnetoelastic film. Amongst other contributions, this noise is closely connected to domain wall activity evoked by the strain from the acoustic waves propagating through the film. A successful method to reduce the presence of domain walls is to couple the ferromagnetic material with an antiferromagnetic material across their interface and therefore induce an exchange bias. In this work we demonstrate the application of a top pinning exchange bias stack consisting of ferromagnetic layers of (Fe_90_Co_10_)_78_Si_12_B_10_ and Ni_81_Fe_19_ coupled to an antiferromagnetic Mn_80_Ir_20_ layer. Stray field closure and hence prevention of magnetic edge domain formation is achieved by an antiparallel biasing of two consecutive exchange bias stacks. The set antiparallel alignment of magnetization provides single domain states over the complete films. This results in a reduction of magnetic phase noise and therefore provides limits of detection as low as 28 pT/Hz^1/2^ at 10 Hz and 10 pT/Hz^1/2^ at 100 Hz.

## Introduction

Sensors for the detection of magnetic fields are essential components in several different areas such as the aerospace and automotive industry, navigation, the security industry or medical diagnostics^1^. In many of these applications the measured signals are artificially generated and the amplitude is either a known threshold value or their angular orientation is of interest^2^. In contrast, very high demands on the detectability are made in biomedical applications which typically exhibit very small amplitude magnetic fields and therefore require a low limit of detection (LOD). Pioneering magnetic measurements of human heart signals have been conducted by David Cohen in the 1960s using a simple coil^3^. Due to obvious limitations in spatial and signal resolution he later switched to more sophisticated methods, taking advantage of the then newly emerging superconducting quantum interference devices (SQUID)^4,5^. This new approach provided a path to measure minimal magnetic fields. However, the search for miniaturized, economical and easy to use alternatives for the SQUID systems has been going on ever since. Different alternative sensor concepts have been proposed to measure small varying magnetic fields, such as optically pumped magnetometers^6,7^, fluxgate magnetometers^8,9^, sensors based on magnetoresistive effects^10,11^ or magnetoelectric composites^12,13^. All of them having their own advantages and disadvantages regarding detection limit, frequency bandwidth, measurement range, spatial resolution, power consumption, lifetime and the necessity for magnetic shielding. All these criteria and the performance of the sensor system as a whole have to be considered to estimate its true capability for biomagnetic diagnostics^14^ or magnetic field-assisted medical applications such as magnetic nanoparticle mapping^15^, active motion sensing^16^ or deep brain stimulation electrode localization and rotational orientation detection^17^.

A magnetic field sensor based on surface acoustic waves (SAW) was first proposed in 1975^18^. However, compared to other sensor concepts such as magnetoresistive sensors, only few research groups have considered this approach^19–22^. SAW magnetic field sensors have only recently gained interest as magnetometers for minimal magnetic fields through the combination of Love wave devices with amorphous magnetostrictive thin films^23^. Their operation principle is based on the generation of high frequency acoustic waves on a piezoelectric substrate by interdigital transducers (IDTs). Using specific cuts of the piezoelectric single crystal substrates in combination with a guiding layer of lower acoustic wave velocity leads to the generation of Love waves^24^. The larger the difference of the mechanical properties between the substrate and the guiding layer is, the stronger is the confinement of the acoustic wave at the guiding layer’s surface^25^. This confinement has the advantage compared to other wave modes such as Rayleigh waves, that influences on the sensor’s surface have a larger impact on the propagating acoustic waves. Such influences can be magnetic fields if the SAW devices are coated with a magnetoelastic film, hence enabling magnetic field sensing capabilities. The sensing principle is based on the delta-E effect, which describes the non-linear change of elastic moduli with magnetization in a magnetostrictive material due to the presence of magnetostrictive strain in addition to the conventional elastic strain of the material^26^. The effective stiffness change changes the velocity of the acoustic waves and leads to a phase shift of the output signal. This change in phase is then proportional to the measured magnetic field amplitude. Different materials and SAW designs have been proposed^27,28^, even solely thin film based SAW magnetic field sensors on silicon wafers have been demonstrated^29^. Particularly high sensitivities can be reached by applying magnetically soft magnetostrictive films with a well-aligned magnetic anisotropy and with a low anisotropy energy density *K*_u_^30^. Their large frequency bandwidth^31^ makes delay line SAW sensors also promising for the localization and rotational orientation detection of implanted deep brain stimulation electrodes^17^.

In SAW based magnetic field sensors the additional noise at low frequencies and low excitation powers has been identified to stem from magnetic losses, which can be expressed as the imaginary part ${\mu }_{\mathrm{r}}^{\mathrm{^{\prime}}\mathrm{^{\prime}}}$ of the complex permeability^32^. These losses are associated with characteristic 1/*f* flicker phase noise and depend on the magnetic domain and anisotropy configuration, the magnetic bias field *H*_DC_ and the power *P*_SAW_ (i.e. the strain acting on the magnetic material) which the SAW sensor is excited with. It was shown that there are complex and manifold interactions between the propagating Love waves and the domain walls within the magnetostrictive film giving rise to potential phase fluctuations leading to noise^33^. Also, for different types of magnetic field sensors such as giant magnetoimpedance (GMI)^34^ or giant magnetoresistance (GMR)^35,36^ based devices it was shown that domain wall activated processes lead to 1/*f* low frequency noise. Additional losses can occur specifically in SAW based sensors due to domain wall resonances which are in the frequency range of typical SAW devices^37^. In general, magnetization noise, or more precise the power spectral density of thermally excited magnetization fluctuations *S*_M_ is directly proportional to the effective magnetic losses ${\mu }_{\mathrm{r}}^{\mathrm{^{\prime}}\mathrm{^{\prime}}}$ according to the fluctuation–dissipation theorem and is given by

1 $${S}_{\mathrm{M}}\left(f\right)= \frac{4{k}_{B}T}{2\pi fV}\frac{{\mu }_{\mathrm{r}}^{\mathrm{^{\prime}}\mathrm{^{\prime}}} (f)}{{\mu }_{0}},$$

where *k*_B_ is the Boltzmann constant, *T* the absolute temperature, *f* the offset frequency, *V* the magnetic volume and *µ*_0_ vacuum permeability^32,38^. Taking the change of magnetization during SAW operation into account the more applicable and measurable power spectral density of phase fluctuations *S*_φ_ can be derived, which is

2 $${S}_{\mathrm{\varphi }}\left(f\right)={S({H}_{\mathrm{DC}},{P}_{\mathrm{SAW}})}^{2}\frac{4{k}_{\mathrm{B}}T}{2\pi fV}\frac{{\mu }_{0}{\mu }_{\mathrm{r}}^{\mathrm{^{\prime}}\mathrm{^{\prime}}}(f{,H}_{\mathrm{DC}}, {P}_{\mathrm{SAW}})}{{(\mu }_{\mathrm{r}}^{\mathrm{^{\prime}}}(f{,H}_{\mathrm{DC}},{P}_{\mathrm{SAW}}){)}^{2}},$$

with *S* being the phase change with change of applied magnetic field, i.e. the sensor’s sensitivity and ${\mu }_{r}^{^{\prime}}$ the real part of the complex permeability^32^.

1/*f*-type noise in magnetic film-based magnetic field sensors is significantly influenced by fluctuations due to magnetic domain wall processes. Therefore, it is desirable to eliminate domain walls in the magnetic films to improve the sensors’ performance. One way to achieve this is by biasing a ferromagnetic material by a coupled antiferromagnetic material. This type of exchange interaction is termed exchange bias^39^. It is associated with a shift of the magnetization loop against an exchange bias field *H*_EB_ which is given by

3 $${H}_{EB}=\frac{{J}_{EB}}{{M}_{S}{t}_{FM}{\mu }_{0}},$$

with the exchange bias energy density *J*_EB_, the saturation magnetization *M*_S_ and the thickness of the ferromagnetic layer *t*_FM_^40^. Especially for spintronic devices such as GMR recording heads the biasing of one of the two ferromagnetic layers by an antiferromagnet leads to a large improvement in device performance through the increase in sensitivity^40–42^. In GMR as well as tunnel magnetoresistance (TMR) devices exchange bias is used for the pinning of one of the ferromagnetic layers of the synthetic antiferromagnet^43,44^. In magnetoelectric (ME) composites exchange bias has been successfully applied for shifting the maximum of the magnetoelectric coefficient to zero field, eliminating the need of an external bias field^45^. Additionally, exchange biasing of ferromagnetic layers can be utilized to reduce magnetic noise in ME composites caused by domain wall nucleation, movement, and annihilation^46,47^, which could be further improved by an antiparallel biasing of consecutive layers^48^. The only two exchange bias system applied to SAW devices so far are Co/MnIr as the IDT material^49^ and CoFeB/MnIr^50^. In ref.^ 49^ the authors emphasize the importance of hysteresis and magnetization state for sensor operation. In contrast to the presented study, the exchange biased sensors were operated out-of-plane and dedicated for high field sensing. In ref.^50^, the device under investigation is not aimed for magnetic field sensing. However, the goal of both studies was not to achieve noise suppression.

On piezoelectric substrates elevated temperatures cause in-plane uniaxial stresses in the magnetostrictive film due to the anisotropic expansion of the substrate. This stress leads to high anisotropy fields *H*_K_ and consequently to a severe reduction of sensor performance^30^. Therefore, the exchange bias in this study is only induced by an external magnetic field applied during the growth of the film stack and the sample is rotated by 180° inside the magnetic field after the deposition of a single layer (SL) stack to achieve antiparallel (AP) exchange bias.

## Results and discussion

### SAW device and exchange bias system

**Figure 1:**
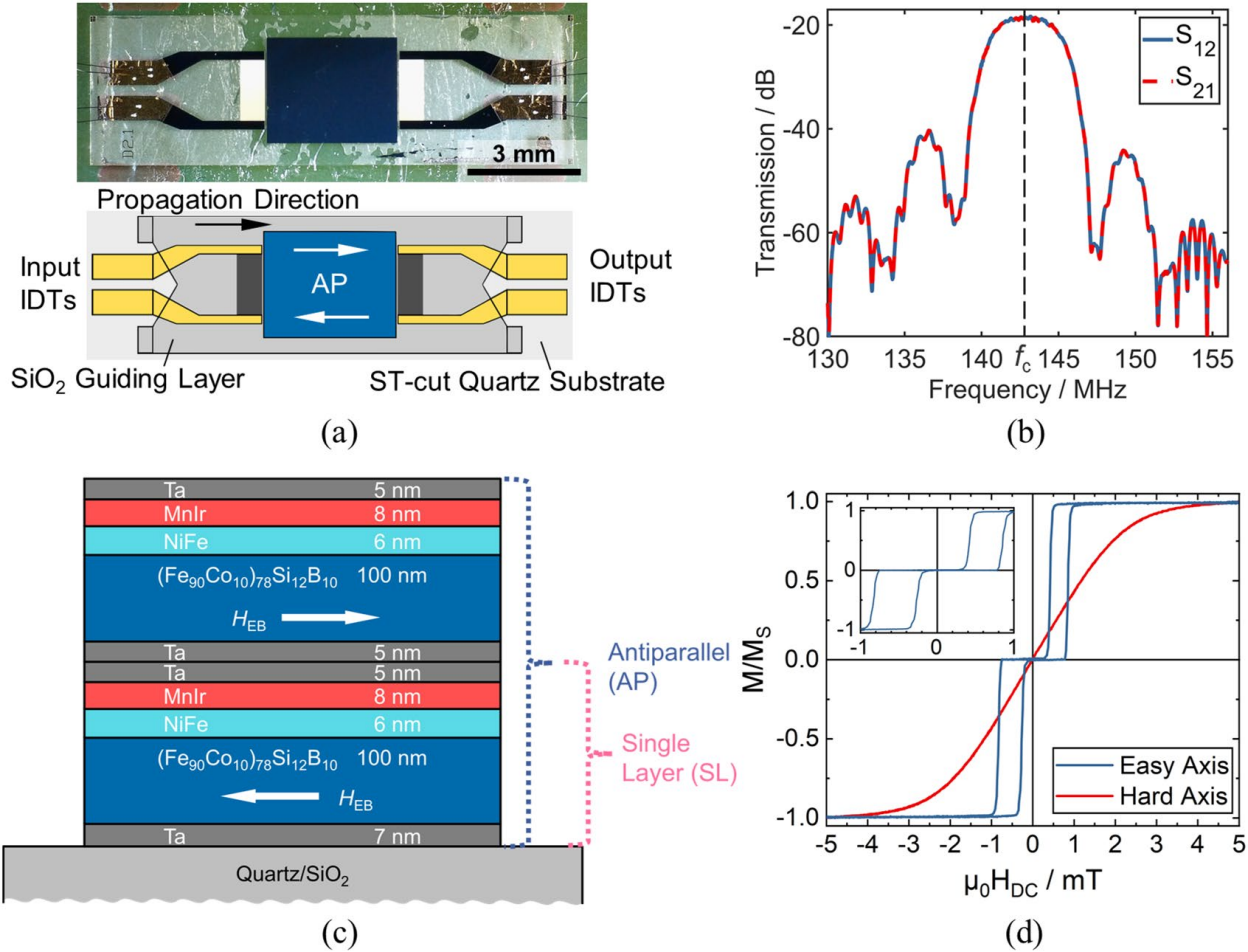
SAW device and exchange bias film stack. (**a**) Top-view photograph and schematic of the Love wave device with a 4 µm SiO_2_ guiding layer. The device is mounted on a PCB with pressure sensitive tape and connected to that PCB by wire bonding. (**b**) Scattering parameters S_12_ and S_21_ of the device showing a synchronous frequency of *f*_c_ = 142.6 MHz and an insertion loss at that frequency of − 18.5 dB. The sensor was magnetically saturated perpendicular to the propagation direction. (**c**) Antiparallel top-pinning exchange bias stack. (**d**) Magnetization loops of the AP exchange bias stack recorded by a BH loop tracer along the easy (blue) and hard axis (red) of magnetization of a circular sample of diameter *d* = 13.8 mm on a ST-cut quartz substrate. The inset shows the easy axis loop in the range of − 1 mT to 1 mT.

A photograph and a schematic of the SAW device under investigation is shown in Fig. 1a. All samples are based on ST-cut quartz with the propagation direction 90° to the crystallographic X-axis, along which shear horizontal waves are excited^24^. The 200 nm thick Au split finger IDTs consist each of 25 finger pairs with a width of 3.5 µm and spacing of 3.5 µm, which in total creates a pitch distance and therefore an acoustic wavelength of λ = 28 µm. Adhesion layers of 8 nm Cr are beneath and above the Au. The acoustic aperture i.e. the width of the acoustic wave front corresponds to 60λ. The measured scattering parameters S_21_ and S_12_ of the sensor under investigation after impedance matching are shown in Fig. 1b. It is magnetically saturated perpendicular to the SAW propagation direction. The sensor exhibits a synchronous frequency of 142.6 MHz and an insertion loss in magnetic saturation of − 18.5 dB. Both are determined mainly by the SiO_2_ guiding layer’s thickness, which is 4 µm in this case and guiding layer’s mechanical properties with respect to the substrate. The exchange bias stack consisting of two sequences of Ta/(Fe_90_Co_10_)_78_Si_12_B_10_/Ni_81_Fe_19_/Mn_80_Ir_20_/Ta is shown in Fig. 1c. Here, the NiFe layer serves as a seed for the antiferromagnetic MnIr (see section “Structural characterization”). The FeCoSiB layers are biased in opposite direction i.e. antiparallel to achieve flux closure and hence prevent the formation of closure domains. As the sample is removed from vacuum after the first deposition step the top Ta layer oxidizes in air. Hence, to still provide adhesion for the FeCoSiB of the second deposition step an additional Ta layer is deposited on the oxidized Ta. The topmost Ta layer acts as passivation layer for MnIr. The magnetization loops of the antiparallel exchange bias stack along the easy (parallel to propagation direction) and hard axis (perpendicular to propagation direction) of magnetization are shown in Fig. 1d. The magnetic field during deposition was also applied 90° to the crystallographic X-axis. Antiparallel shifts of the easy axis hysteresis loop can be observed corresponding to exchange bias fields of *µ*_0_*H*_EB,l_ = 0.5 mT (l for left hand side shift) and *µ*_0_*H*_EB,r_ = − 0.6 mT (r for right hand side shift). In comparison to samples with similar thicknesses of the ferromagnetic and antiferromagnetic layers, but in which the bottom-pinned exchange bias was induced by annealing in a magnetic field, our samples show an about four times smaller exchange-bias strength^48^. However, for the system Ni_81_Fe_19_/Mn_78_Ir_22_ the exchange bias energy is, according to (3), *J*_EB,NiFe_ = 70 µJ/m^2^^51^, which is comparable to this system with each stack exhibiting coupling energies of *J*_EB,l_ = 61 µJ/m^2^ and *J*_EB,r_ = 73 µJ/m^2^, respectively with *M*_S_ = 1.45 T. The coercivity fields of the two hysteresis loop branches are *µ*_0_*H*_C,l_ = 0.27 mT and *µ*_0_*H*_C,r_ = 0.22 mT, respectively. This only small difference in coercivity could be explained by a slight tilt of the anisotropies of the two layers with respect to each other. Along the hard axis of magnetization no measurable hysteresis is observed. The anisotropy field of this system on ST-cut quartz is found to be *µ*_0_*H*_k_ = 1.7 mT which is only slightly higher than in non-exchange biased FeCoSiB with 1.5 mT ^30^ and the total anisotropy field, which is the sum of *H*_k_ and *H*_EB_ is *µ*_0_*H*_k,tot_ = 2.3 mT.

### Structural characterization

**Figure 2:**
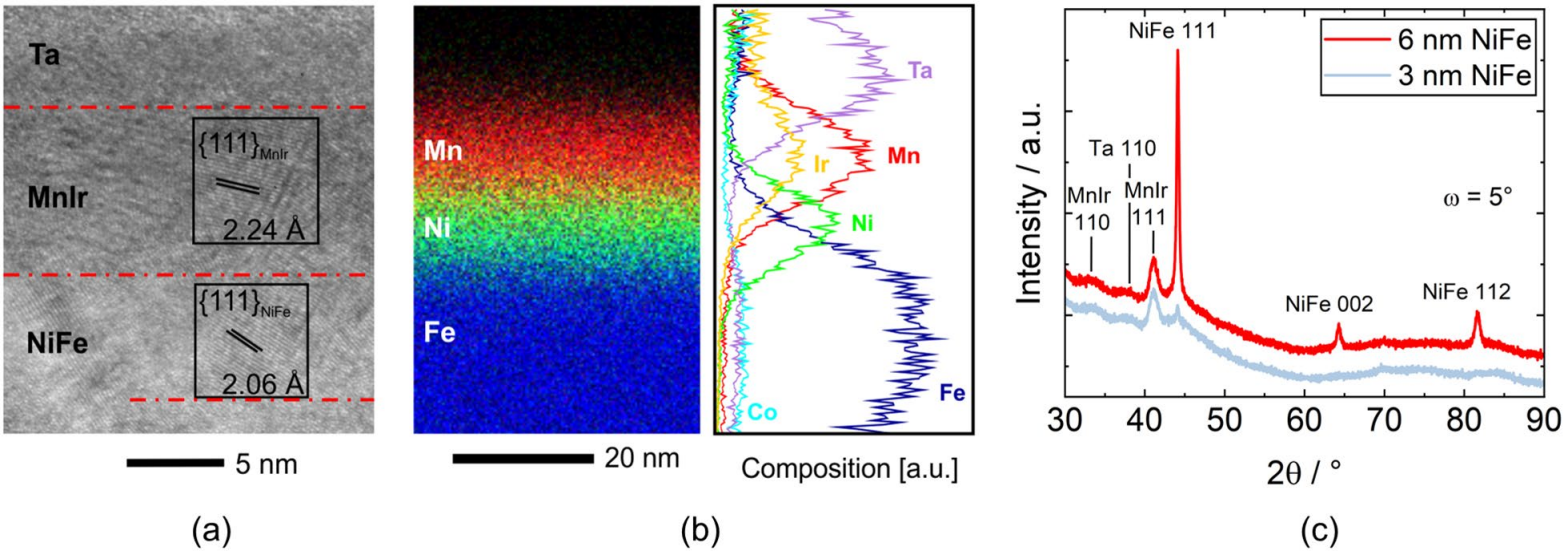
TEM investigation and X-ray diffraction of a section of a SL exchange bias stack. (**a**) High resolution TEM image of a single exchange bias stack with 6 nm NiFe. (**b**) EDS elemental map and quantified profiles across the functional top layers. (**c**) Diffractograms of the SL stack with two different NiFe thicknesses. The incident angle was kept constant at ω = 5° to only penetrate the thin films and not the single crystal substrate.

Figure 2a shows a noise-filtered high-resolution TEM micrograph from a section of a single layer exchange bias stack. The FeCoSiB layer is amorphous as intended, only at the interface to NiFe crystalline regions are observed. However, at the interface a clear distinction between the two layers is not possible. The layers of NiFe and MnIr are polycrystalline containing nanosized grains with distinct {111} texture along the growth direction. Localized elemental analysis by energy-dispersive X-ray spectroscopy (EDS) mapping in Fig. 2b is conducted for validation of the layer thicknesses and the elemental distribution across the stack, e.g. to examine potential intermixing. Mostly, the average thicknesses of all functional layers matched with the anticipated target thickness, considering that there are no sharp transitions between the layers which limits the resolution. This smearing of the elemental distribution across the interfaces is apparent from the elemental map, showing exemplary Mn, Ni and Fe signals, as well as the quantified profiles of all metallic elements averaged across the mapped region. This feature is especially prominent for the broad and diffuse (Fe_90_Co_10_)_78_Si_12_B_10_/Ni_81_Fe_19_ interface, featuring the apparent decrease of Fe content in the NiFe layer. However, as the stoichiometry of the NiFe layer should be 81:19, the observed Fe profile can be interpreted as the overlap of the extended signal scattering background from Fe in the FeCoSiB layer and a Gaussian-profile of Fe in Ni_81_Fe_19_. Noteworthy, a significant degree of roughness at the NiFe/MnIr interface is observed from high-resolution investigations, which could rationalize the overlap of localized X-ray intensities, as well as sample thickness, which leads to X-ray signal delocalization. It was shown that roughness can have an influence on exchange bias and coercivity fields^52^.

For sufficient and reliable exchange bias when using MnIr as the antiferromagnetic material a {111} texture of the MnIr is beneficial^53^. In order to adjust this texture of MnIr, NiFe is acting as a supporting seed layer. However, the relation between MnIr texture and exchange bias field is complex and even contradicting findings have been reported^54^. The X-ray diffractograms in Fig. 2c reveal that this texture is present with a MnIr 111 reflection at 41.1°. Even using a NiFe thickness of 3 nm the same MnIr 111 reflection intensity is present. In fact, NiFe layers with thicknesses of 3 nm and 6 nm both result in the same exchange bias fields (not shown), but for reproducibility aspects a higher thickness was chosen for this study.

### Sensor response

**Figure 3:**
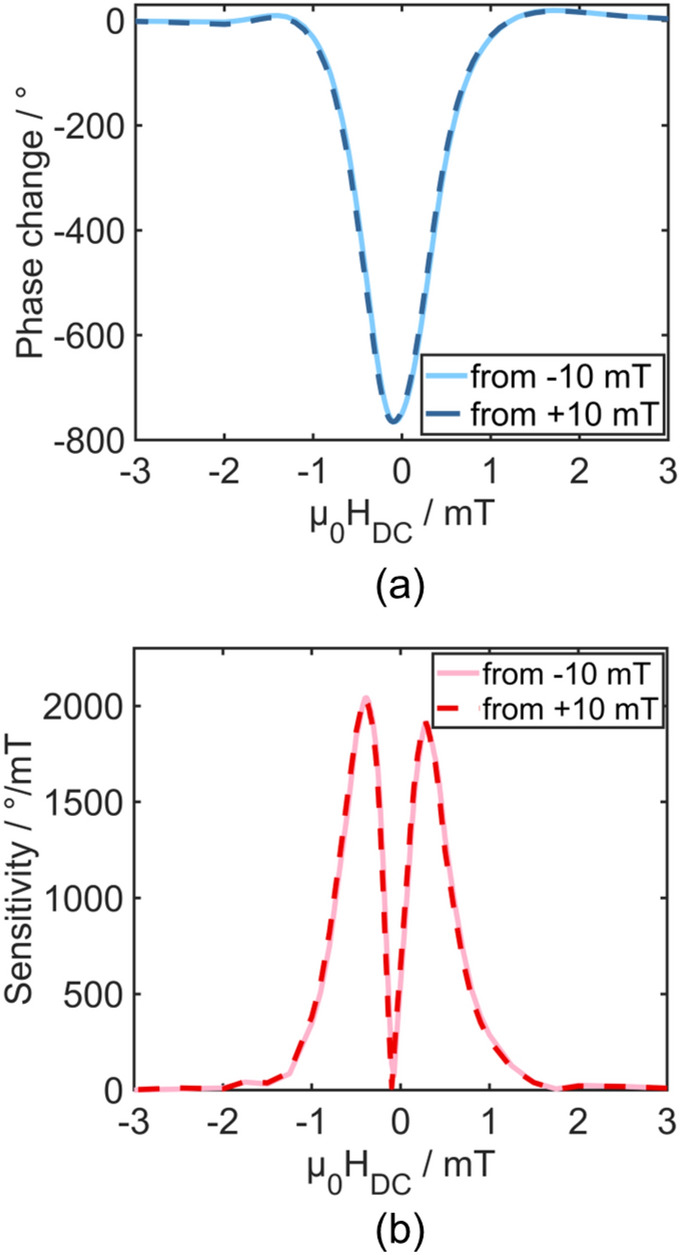
Phase response and sensitivity. (**a**) Phase change as a function of applied DC bias field µ_0_H_DC_ of the Love wave sensor with AP exchange bias. (**b**) The sensor’s measured sensitivity as a function of applied DC bias field. In both measurements the excitation power was 10 dBm (10 mW) and the magnetic field was applied perpendicular to the SAW propagation direction, i.e. the exchange bias axis.

**Figure 4:**
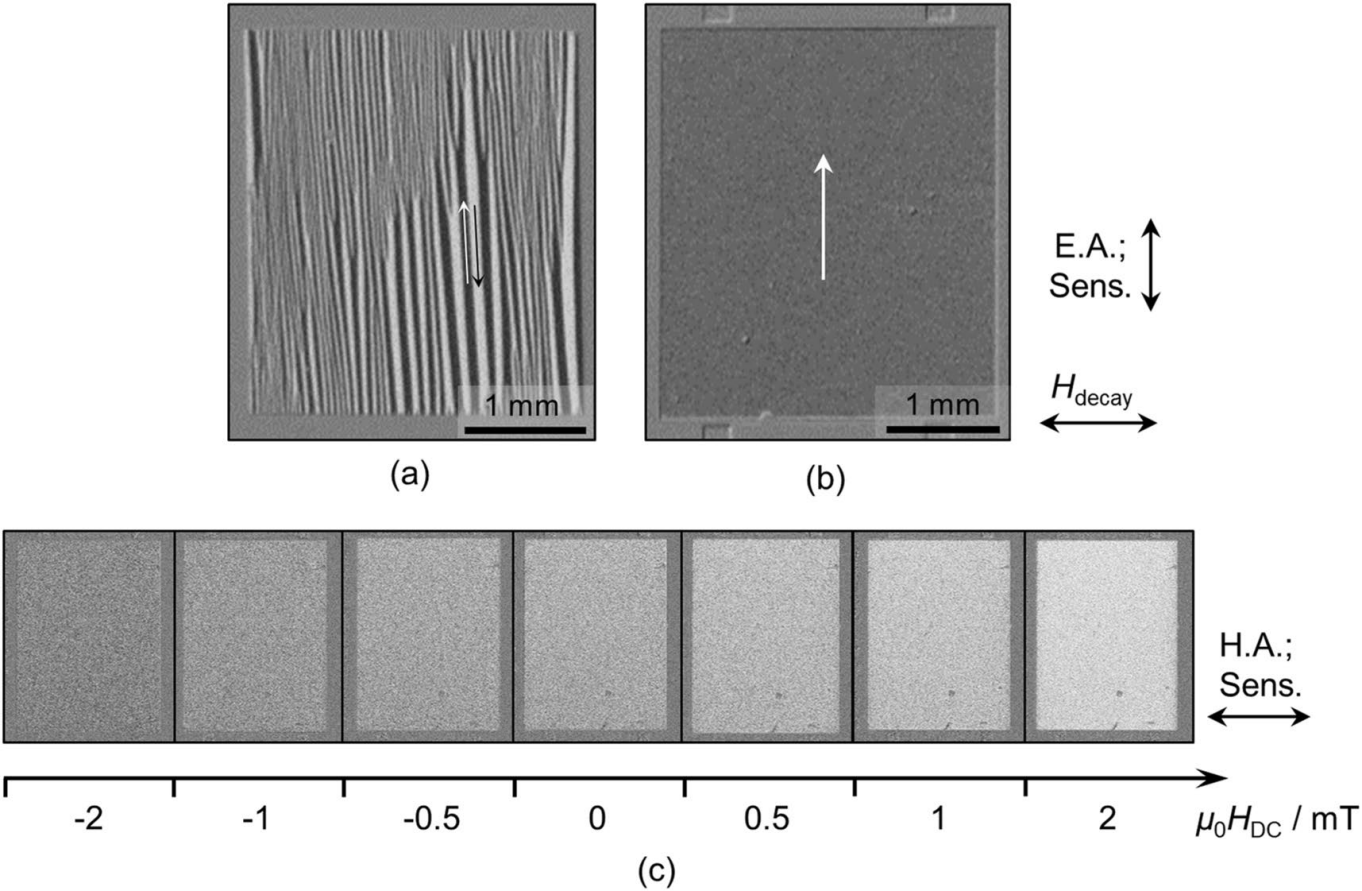
Magneto-optical Kerr effect microscopy images. (**a**) A 200 nm single layer of FeCoSiB. (**b**) The top layer of antiparallel exchange biased 2 × 100 nm FeCoSiB. Both samples have been demagnetized by an decaying AC magnetic field H_decay_ along the hard axis of magnetization. The sensitivity axis of the MOKE microscope was vertically i.e. along the easy axis of magnetization, which is also the SAW propagation direction. (**c**) The top layer of antiparallel exchange biased 2 × 100 nm FeCoSiB in which the applied field was changed stepwise from − 5 to 5 mT (shown are only images from − 2 to 2 mT) along the hard axis of magnetization while also the MOKE sensitivity was set along this axis. The gray area around the FeCoSiB layers is the non-ferromagnetic SiO_2_ layer. In (**b**) also parts of the input and output IDTs can be seen through the SiO_2_ layer.

In the case of delay line SAW magnetic field sensors the sensitivity is the relation of the phase change to the amplitude of an applied magnetic field. In all sensors based on magnetic materials this sensitivity is dependent on the magnetization state of that material which can be altered by an external magnetic DC field. The phase change of a SAW sensor with antiparallel exchange bias as a function of applied DC magnetic field is shown in Fig. 3a. It follows the characteristic change of the shear modulus of a magnetostrictive material under shear SAW excitation where the easy axis magnetization is parallel to the propagation direction and perpendicular to the DC magnetic field^55^. The total phase change between magnetic saturation and the minimum at about − 0.05 mT amounts to 770°. Sweeping the magnetic field from − 10 mT to + 10 mT and vice versa results in very little hysteresis, which indicates a remagnetization process governed dominantly by coherent magnetization rotation rather than domain wall motion (see Fig. 4c). The two curves are shifted with respect to each other by only up to 25 µT. Additionally, the minima of each curve are slightly shifted indicating a small tilt of the magnetic anisotropies with respect to the propagation direction. The SAW sensor’s measured sensitivity is shown in Fig. 3b. It is obtained by applying a 1 µT sinusoidal modulation field of 10 Hz and at each measurement point the phase at 10 Hz is read out and divided by the 1 µT AC field. The sensitivity also represents the derivative of the phase change and therefore the sensitivity maxima in Fig. 3b correspond to the points of highest slope in Fig. 3a. These maxima are at − 0.4 mT and at 0.28 mT with sensitivities of 2040 °/mT and 1920 °/mT, respectively. In an application specific operation these bias fields can be provided by e.g. permanent magnets with defined remanent magnetization and distance to the sensor. Exchange bias has also been incorporated in magnetoelectric composites to provide intern bias shifting the maxima of the piezomagnetic coefficient to zero field^45^. Whether this approach is applicable to SAW magnetic field sensors requires further investigation. Nevertheless, despite a lower hard axis magnetic permeability than comparable SAW devices based on the same magnetostrictive material and same thickness but without exchange bias, this work’s sensors show similar sensitivities^30^.

### Magneto-optical imaging

The goal of exchange biasing the FeCoSiB layers is to achieve a single domain state in the magnetic thin film. Stacking two or more magnetic flux compensating exchange biased layers with antiparallel aligned magnetization reduces the total demagnetizing field energy of the sample and therefore suppresses the formation of closure domains^48^. In the case of complete closure of magnetic flux, a single domain state at remanence can be achieved. To verify that single domain state of FeCoSiB in the antiparallel exchange bias stack magneto-optical Kerr effect (MOKE) microscopy imaging has been conducted. Figure 4 shows a comparison of the magnetic states of 200 nm plain FeCoSiB (Fig. 4a) and a 2 × 100 nm antiparallel exchange bias sample (Fig. 4b). In Fig. 4b only the top layer is visible, which is additionally covert by the MnIr and NiFe layers leading to less MOKE contrast in this image. Before both images were taken the magnetic film was demagnetized by an AC magnetic field with decaying amplitude of which the initial amplitude was high enough to saturate the films. The demagnetizing field *H*_decay_ was applied along the hard axis of magnetization to achieve a magnetic ground state. Without exchange bias thin straight domains are formed with a high domain wall density. Additionally, characteristic closure domains form at the edges^56^. In contrast, the top layer in the antiparallel biased system shows a single domain state. This is underlined in Fig. 4c, which shows MOKE contrast images of the same antiparallel exchange biased sensor, but in this *µ*_0_*H*_DC_ was changed from − 5 to 5 mT (shown are images from − 2 mT to 2 mT) along the hard axis of magnetization. This is the same axis as in the hard axis loop in Fig. 1d and in the bias curves in Fig. 3. No magnetic domains form during the remagnetization process without excitation, confirming the anticipated coherent magnetization rotation in the structured sensor.

### SAW sensor performance

**Figure 5:**
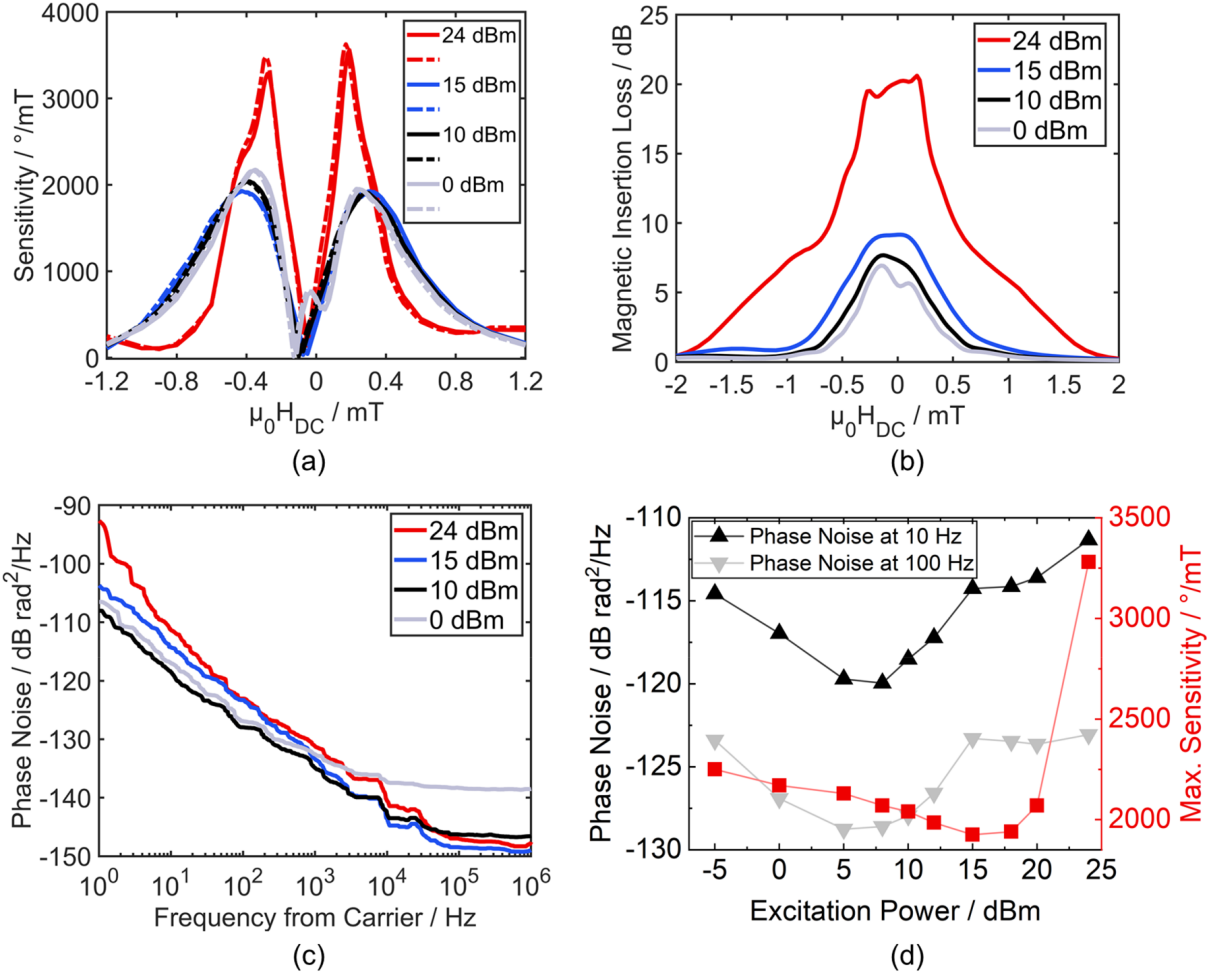
Power dependency of the antiparallel exchange biased SAW sensor’s performance. (**a**) Sensitivity as a function of applied magnetic DC bias field swept from − 10 mT to + 10 mT (full lines) and vice versa (dashed lines) for different excitation powers. Only the region of high sensitivity from − 1.2 mT to + 1.2 mT is shown. (**b**) Magnetically induced insertion loss as a function of applied DC magnetic field for different excitation powers measured from − 10 mT to + 10 mT. The losses are normalized to 0 dB, where 0 dB then corresponds the insertion loss in magnetic saturation. (**c**) Phase noise spectra of the same sensor at different excitation powers. For every excitation power a DC bias field was applied which corresponds to the point of highest sensitivity. (**d**) Phase noise at 10 Hz and 100 Hz and the respective maximum sensitivity as a function of excitation power.

For a single magnetic layer SAW device it was shown that, in general, with increasing excitation power the flicker phase noise in SAW magnetic field sensors is decreasing, as also the effective losses, i.e. ${\mu }_{\mathrm{r}}^{\mathrm{^{\prime}}\mathrm{^{\prime}}}$ are decreasing^32^. However, this only holds true up to a certain excitation power. With increasing excitation random Barkhausen domain wall jumps occur, which at high enough excitation power amplitudes become the dominant source of noise, causing so called random walk of phase noise exhibiting 1/*f*^2^ behavior. Consequently, the SAW sensors’ excitation power dependency in a single domain system is investigated. First, Fig. 5a shows the sensitivity of an antiparallel exchange biased sensor as a function of DC bias field for different excitation powers. It shows that even applying powers as high as 15 dBm the magnetic field dependency does not change significantly. Typical excitation powers for SAW magnetic field sensors are around 0 dBm^32^. In fact, with increasing power the sensitivity curves even become more symmetric, i.e. the sensitivity values at the maxima become equal and the peaks of maximum sensitivity slightly shift to higher fields. Both effects can be explained by the additional effective magnetic anisotropy caused by the oscillating shear stress. However, with severely higher excitation powers the shape of the sensitivity curves changes. Apart from the main maxima at small magnetic field values an additional change in shape as small “humps” occur at around − 0.4 mT/0.4 mT. The drastic increase in sensitivity at smaller magnetic bias fields suggests an altered remagnetization mechanism with higher magnetic permeability which is not present as significantly at lower powers. For visualization the maximum sensitivities are plotted against different excitation powers in Fig. 5d.

In Fig. 5b magnetically induced additional insertion losses relative to the insertion losses in magnetic saturation at the respective excitation powers are shown as a function of applied DC bias fields for different excitation powers. The additional magnetic insertion losses show a maximum at around zero magnetic field for all excitation powers. Analogous to the sensitivity in Fig. 5a also the losses first become more symmetric around zero magnetic field and strongly increase at high excitation amplitudes. In previous studies magnetic insertion losses have been connected with the increased presence of domain walls^33^. A clear correlation between additional magnetic insertion loss and magnetically induced phase noise has also been observed^32^. There in a simple layer of FeCoSiB phase noise is highest where the insertion loss is maximum at a constant excitation power. Comparing Fig. 5a and b shows that at lower powers the sensitivity maxima are not correlating with high insertion losses. Only at high powers such as 24 dBm the sensitivity maxima correspond to the two minor peaks in magnetic insertion loss at − 0.28 mT and 0.19 mT in Fig. 5b. Here, Fig. 5c reveals that the phase noise is only correlating with magnetic insertion losses to a certain degree. It shows the power spectral density of phase fluctuations i.e. the phase noise as a function of frequency next to the excitation carrier for different excitation powers. While the insertion losses increase continuously with excitation power, the phase noise first decreases and then increases again. This is visualized in Fig. 5d showing the phase noise at 10 Hz and 100 Hz as a function of excitation power. It clearly reveals a region where the phase noise is minimized, which is between 5 and 10 dBm. Compared to non-exchange biased FeCoSiB-based SAW sensors higher excitation powers can be applied before the noise increases^32^. Additionally, the noise increase is not as drastic with higher powers, suggesting other noise source mechanisms rather than random Barkhausen jumps. Therefore, compared to non-exchange biased SAW sensors the phase noise at an optimal working point is 8 dB lower^30^. One potential noise source is the temperature generated during SAW excitation with elevated power^57,58^. In our devices we found an increase of temperature of 14 °C from room temperature at an excitation power of 24 dBm (not shown). According to Eq. (2) this will lead to an increase of 1/*f* phase noise. Additionally, the oscillating shear strain from the acoustic wave can potentially cause magnetization fluctuations in the film leading to noise. In^33^ the shear stress generated by the wave amplitude was estimated from the change in MOKE contrast to be *τ* = 3.29 MPa at 10 dBm excitation. Assuming a shear modulus of FeCoSiB of *G* = 28.1 GPa^59^ the acting shear strain at 10 dBm excitation can be estimated as *γ*_10dBm_ = 0.06‰. At 24 dBm the excitation amplitude is about 5 times higher leading to estimated shear strains of *γ*_24dBm_ = 0.3‰ under the assumption that the strain is increasing linearly with excitation amplitude, which is proportional to the square root of the excitation power. In the case of Love waves the oscillating shear strain is acting 45° with respect to the magnetic easy axis and triggers an oscillation of the magnetization. However, the specific mechanisms require further investigation.

**Figure 6:**
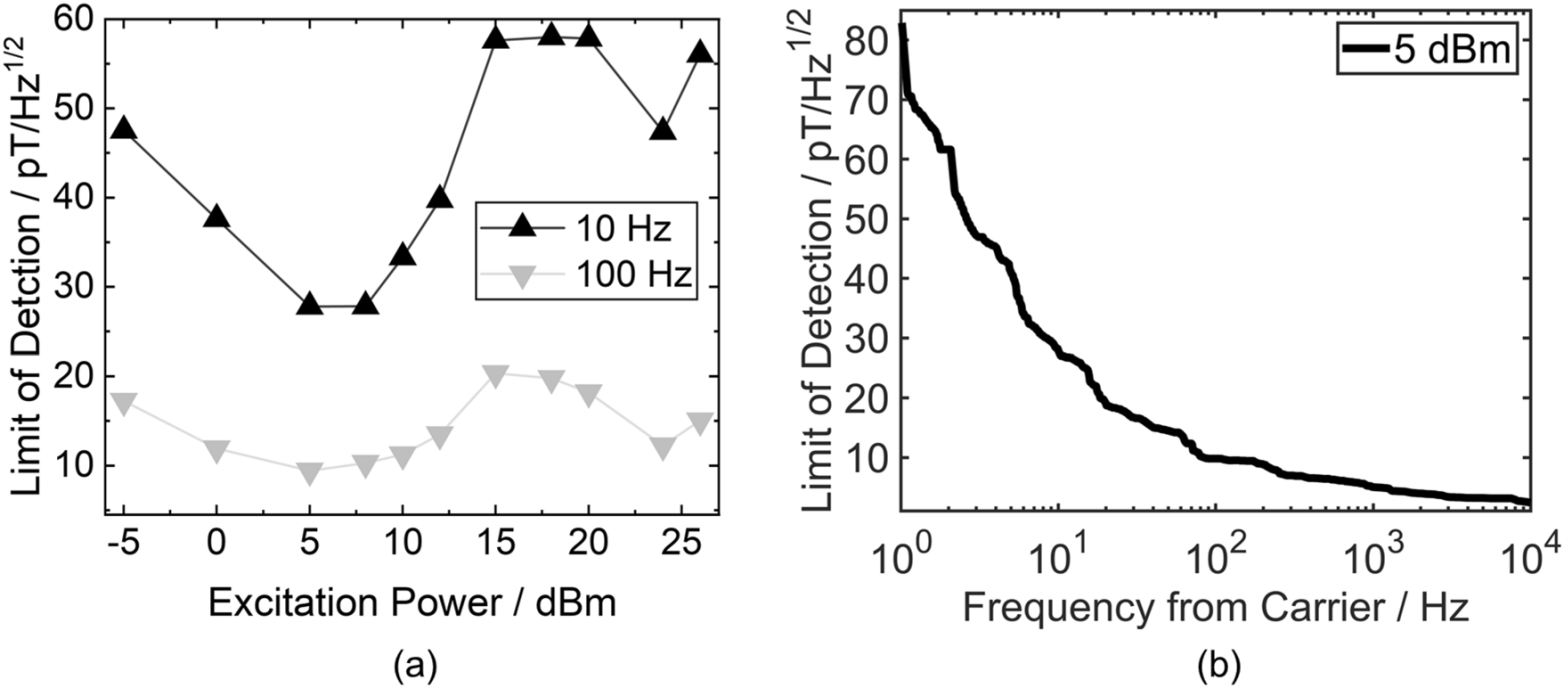
Limits of detection (LOD). (**a**) LOD as a function of excitation power at 10 Hz and 100 Hz off the carrier frequency. (**b**) Frequency spectrum of the lowest LOD at 5 dBm excitation power.

The limit of detection (LOD) which is the ratio of phase noise and sensitivity^23^ is shown in Fig. 6a for different excitation power at 10 Hz and 100 Hz. Since the sensitivity is barely changing up to excitation powers of 18 dBm the LOD follows the same trend as the phase noise, with the lowest LODs between 5 and 8 dBm of 28 pT/Hz^1/2^ at 10 Hz and 10 pT/Hz^1/2^ at 100 Hz. This is an improvement by a factor of 2.5 compared to the very best non-exchange biased SAW sensors so far^30^. At higher excitation powers from 15 dBm on the LOD is almost constant as the noise increases in the same amount as the sensitivity. The lowest LOD at 5 dBm excitation is shown in Fig. 6b up to a frequency of 10 kHz, as in this range the sensitivity of the sensor is constant^31^. From 1 kHz on the LOD is even below 5 pT/Hz^1/2^.

## Conclusions

A top pinning exchange bias stack consisting of ferromagnetic layers of Ni_81_Fe_19_ and magnetostrictive (Fe_90_Co_10_)_78_Si_12_B_10_ and antiferromagnetic Mn_80_Ir_20_ on Love wave SAW devices was presented. An antiparallel biasing of two exchange bias stacks was achieved by applying a magnetic field during deposition and by rotating the sample between depositions. The NiFe seed layer provides a 111 texture of the MnIr layer, inducing an exchange bias which is high enough to provide a single domain state over the complete magnetic film. The phase response of the SAW sensors exhibits small hysteresis and due to the elimination of domain walls the magnetic 1/*f* phase noise was reduced by about 8 dB compared to non-exchange biased sensors. However, it was also shown that despite the likely absence of domain walls, there is an excitation power dependency of the phase noise. At high excitation power amplitudes 1/*f* noise increases which makes further investigations necessary to distinguish between different noise contributions. Overall, a substantial improvement of the limit of detection of SAW magnetic field sensors by a factor of 2.5 was achieved.

## Methods

### Sample and device fabrication

Au IDTs of 200 nm thickness with 8 nm Cr adhesion layers on top and bottom are deposited by DC magnetron sputtering and structured by photolithography and ion beam etching. Subsequently, a 4 µm thick SiO_2_ layer is deposited by means of plasma-enhanced chemical vapor deposition (PECVD) acting as a guiding layer. Parts of the SiO_2_ are removed again by inductively coupled plasma reactive ion etching to provide access to the contact pads for wire bonding of the final sensor to a printed circuit board (PCB). The deposition of the antiparallel top pinning exchange bias stack is performed in two steps. First, layers of Ta (7 nm)/(Fe_90_Co_10_)_78_Si_12_B_10_ (100 nm)/Ni_81_Fe_19_ (6 nm)/Mn_80_Ir_20_ (8 nm)/Ta (5 nm) are deposited while a magnetic field of ~ 60 mT is present which is applied parallel to the SAW propagation direction. Afterwards, the samples are removed from the vacuum chamber, rotated by 180° with regards to the magnetic field and a second sequence of layers is deposited consisting of Ta (5 nm)/FeCoSiB (100 nm)/NiFe (6 nm)/MnIr (8 nm)/Ta (5 nm). For magnetic and structural characterization single layer (SL) stacks have been fabricated as well with 6 nm and 3 nm thick NiFe. The depositions of FeCoSiB and NiFe are done by RF magnetron sputtering and of Ta and MnIr by DC magnetron sputtering. The geometrical delay line structure of the exchange bias layers is provided via lift-off.

### Structural characterization

A cross-section of a single exchange bias stack is prepared by the focused ion-beam (FIB) method and investigated using transmission electron microscopy (TEM). High-resolution micrographs of the NiFe and MnIr crystalline layers are recorded on a Tecnai F30 G^2^ STWIN microscope. Elemental mapping using energy-dispersive X-ray spectroscopy (EDS) of the functional layers is performed in scanning mode on a JEOL NeoARM. X-ray diffractograms are obtained using a Rigaku SmartLab 9 kV X-ray diffractometer with CuK_α_ (λ = 1.5406 Å) radiation.

### Magnetic characterization and MOKE microscopy

Magnetic domain images are gathered with a large view magneto-optical Kerr effect (MOKE) microscope. Homogeneous illumination of the sample is achieved by a combination of a high-power LED source (520 nm wavelength) with a telecentric lens. A Scheimpflug CCD camera mount is used for getting an even focus across the large sample area. Volumetric magnetic hysteresis loops are recorded with an inductive BH loop tracer.

### Sensor characterization

The sensor is wire bonded to a PCB and its impedance is matched to 50 Ω on each port. Scattering parameters are measured with a vector network analyzer while the sensor is magnetically saturated perpendicular to the SAW propagation direction. Magnetic sensor characterizations are performed within a zero-gauss-chamber to eliminate influences from earth’s magnetic field and surrounding laboratory equipment. DC and AC magnetic fields are provided by two solenoids, respectively, while for dynamic phase detection a 1 µT sinusoidal AC signal of 10 Hz is applied. In the experiments all magnetic fields are applied along the hard axis of magnetization, i.e. perpendicular to the SAW propagation direction. For excitation and read out a Zurich Instruments UHFLI lock-in amplifier is used. To apply higher power amplitudes than the 7.5 dBm maximum power provided by the UHFLI a ZFL − 2500VH + power amplifier from Mini Circuits is. The ZFL power amplifier provides a gain of 24 dB and has a noise level of − 138 dB rad^2^/Hz at 10 Hz and − 152 dB rad^2^/Hz at 1 kHz when applying 0 dBm. For noise measurements a Rohde&Schwarz FSWP phase noise analyzer is used while the same ZFL-2500VH + power amplifier provides higher power amplitudes and a step attenuator smaller power step sizes. As a source for the DC bias fields during noise measurements an in-house built battery-based potentiometer-controlled low-noise source is used.

## Data Availability

The data that supports the findings of this study are available from the corresponding author upon request.
